# Low-dose immunogenic chemotherapeutics promotes immune checkpoint blockade in microsatellite stability colon cancer

**DOI:** 10.3389/fimmu.2022.1040256

**Published:** 2022-10-27

**Authors:** Yuhang Fang, Haoyu Sun, Xinghui Xiao, Maoxing Tang, Zhigang Tian, Haiming Wei, Rui Sun, Xiaodong Zheng

**Affiliations:** ^1^ Hefei National Research Center for Physical Sciences at Microscale, the CAS Key Laboratory of Innate Immunity and Chronic Disease, School of Basic Medical Sciences, Division of Life Sciences and Medicine, University of Science and Technology of China, Hefei, China; ^2^ Institute of Immunology, University of Science and Technology of China, Hefei, China

**Keywords:** TIGIT blockade, immunogenic chemotherapeutics, immunogenic cell death, colon cancer, microsatellite stability

## Abstract

More than 85% of colorectal cancer (CRC) patients, who are with microsatellite stability (MSS), are resistant to immune checkpoint blockade (ICB) treatment. To overcome this resistance, combination therapy with chemotherapy is the most common choice. However, many CRC patients do not benefit more from combination therapy than chemotherapy alone. We hypothesize that severe immunosuppression, caused by chemotherapy administered at the maximum tolerated dose, antagonizes the ICB treatment. In this study, we found that low-dose oxaliplatin (OX), an immunogenic cell death (ICD)-induced drug, increased the antitumor response of TIGIT blockade against CT26 tumor, which is regarded as a MSS tumor. Combined treatment with OX and TIGIT blockade fostered CD8^+^ T-cell infiltration into tumors and delayed tumor progression. Importantly, only low-dose immunogenic chemotherapeutics successfully sensitized CT26 tumors to TIGIT blockade. In contrast, full-dose OX induces severe immunosuppression and impaired the efficacy of combination therapy. Further, we also found that lack of synergy between nonimmunogenic chemotherapeutics and TIGIT blockade. Consequently, this study suggests that the strategies of combination treatment of chemotherapy and ICB should be re-evaluated. The chemotherapeutics should be chosen for the potential to ICD and the dosage and regimen should be also optimized.

## Introduction

Colorectal cancer (CRC) is the third highest incidence tumor and the second leading cause of cancer-related deaths in the world (1). The prognosis of patients with CRC remains poor, although early detection through screening has improved the outcome of patients with CRC; approximately 20% of patients still present with metastatic cancer (2), and a further one-third present with early-stage disease but go on to develop metastatic disease with a 5-year survival of only 15% (3). Thus, the development of effective treatments for CRC patients is an urgent need.

Numerous studies have revealed that immune checkpoint blockade (ICB) therapy is one of the most successful approaches against various solid tumors, such as melanoma and non-small cell lung cancer (4). In CRC, PD-1 blockade has been approved for the treatment of heavily mutated tumors that are mismatch repair deficient (dMMR) or have microsatellite instability (MSI) (5–7). However, more than 85% of CRC patients (8), which are mismatch repair proficient (pMMR) or have microsatellite stability (MSS), are not able to benefit from PD-1 blockade therapy (9, 10). In these patients, the lack of tumor mutation and immune cell infiltration has been posited as mechanisms of ICB resistance (8, 11–13). It is important to clarify which strategies can be employed for converting tumor microenvironments lacking immune cell infiltration to those displaying antitumor immunity. Therefore, alternative immunotherapies are required for those patients with pMMR/MSS CRC.

Chemotherapy, the most common treatment for CRC (14, 15), is considered in combination with ICB therapy (16, 17). Conventional chemotherapeutic drugs have been identified based on their capacity to prevent the growth of human tumor cells cultured *in vitro* or transplanted into immunodeficient mice without considering the contribution of the immune system (18). According to this strategy, multiple cytotoxic agents are developed as antitumor drugs, which are not restricted to tumor cells but rather to most normal cells, including immune cells (19). Chemotherapies administered at the maximum tolerated dose (MTD) cause severe immunosuppression, such as lymphopenia and myelosuppression (20), which suggests a possible antagonist between chemotherapy and immunotherapy. However, a few drugs, such as oxaliplatin (OX) (21) and doxorubicin (22), are able to cause immunogenic cell death (ICD) and boost antitumor immunity (23, 24). Therefore, we hypothesize that ICD-induced drugs could enhance ICB therapy and overcome the resistance of pMMR/MSS CRC when administered at low or moderate doses.

It has been reported that TIGIT blockade can enhance the infiltration of T cells and NK cells into weakly immunogenic or metastatic tumors (25). Here, we found that low-dose OX, an ICD-induced drug, increased the antitumor response of TIGIT blockade against CT26 tumor, which is regarded as a pMMR/MSS tumor. Combined treatment with OX and TIGIT blockade fostered CD8^+^ T-cell infiltration into tumors and delayed tumor progression. Importantly, only low-dose immunogenic chemotherapeutics successfully sensitized CT26 tumors to TIGIT blockade. In contrast, full-dose OX induces severe immunosuppression. Consequently, this study suggests that chemotherapeutic drugs, which should be rationally selected to enhance tumor immunogenicity, can be used to make resistant tumors sensitive to checkpoint blockade therapy. In addition, the dosage and regimen of combined treatment should be also optimized.

## Methods

### Mice

C57BL/6J and BALB/c mice were purchased from the Shanghai Experimental Animal Center (Shanghai, China). *Rag2^–/–^* mice were provided by Dr. X. Wang (Inner Mongolia University). All mice were maintained in a specific pathogen free facility and used according to the guidelines for experimental animals at the University of Science and Technology of China. Mice were used between 6 weeks and 8 weeks of age.

### Cell lines

The CT26 cell line was purchased from the Cell Bank of the Chinese Academy of Sciences (Shanghai, China). The MC38 cell line was kindly provided by Professor Yangxin Fu from the University of Texas Southwestern Medical Center (Dallas, USA). All cell lines tested negative for mycoplasma contamination.

### Tumor models

BALB/c or *Rag^2–/–^* mice were inoculated subcutaneously with 5 × 10^4^ CT26 cells. C57BL/6J mice were inoculated subcutaneously with 5 × 10^4^ MC38 cells. Eight days later, mice were randomized into different treatment groups and treated with anti-TIGIT (10 mg/kg; purified in-house from 13G6 cell supernatants), oxaliplatin (1.5 or 6 mg/kg; S1224, Selleck), cisplatin (0.25 mg/kg; S1166, Selleck) or isotype-matched control antibody (10 mg/kg; purified in-house from ratserum) by intraperitoneal injection. Tumors were measured every two days by caliper, and tumor volume was calculated as 0.5 × length × width × width.

### Isolation of TILs

TILs were isolated by dissociating tumor tissue in the presence of collagenase IV (0.1% w/v, Sigma) and DNAse I (0.005% w/v, Sigma) for 1 h before centrifugation on a discontinuous Percoll gradient (GE Healthcare, Little Chalfont, UK). Isolated cells were then used in various assays to evaluate the phenotype and function of NK cells and T cells and to calculate their absolute numbers.

### Antibodies and flow cytometry

Monoclonal antibodies to mouse TIGIT were purified in-house from hybridoma cell (13G6) supernatants (25). The isotype-matched control antibodies (rat IgG) were purified in-house from rat serum. Anti-CD8β antibody (53-5.8) was purchased from Bio X Cell (Lebanon, USA). Rabbit anti-ASGM1 was purchased from Wako Pure Chemicals. The following reagents were used: PE-conjugated antibodies to mouse Granzyme B (16G6, eBioscience, San Diego, USA) and FasL (MFL3, BD Pharmingen, San Diego, USA); PerCP-CY5.5-conjugated antibody to mouse CD3ϵ (145-2C11, BioLegend, San Diego, USA), CD49b(DX5, BioLegend, San Diego, USA) and TRAIL (N2B2, BioLegend, San Diego, USA); PE-Cy7-conjugated antibodies to mouse NKp46(29A1.4, eBioscience, San Diego, USA); APC-conjugated antibodies to mouse Perforin (eBioOMAK-D, eBioscience, San Diego, USA); BV421-conjugated antibodies to mouse CD49b(DX5, BD Pharmingen, San Diego, USA) and TNF-α (MP6-XT22, BioLegend, San Diego, USA); BV510-conjugated antibody to mouse CD45(30-F11, BD Pharmingen, San Diego, USA); BV605-conjugated antibody to mouse CD3ϵ (145-2C11, BD Pharmingen, San Diego, USA); BV786-conjugated antibody to mouse IFN-γ (XMG1.2, BD Pharmingen, San Diego, USA); BUV395-conjugated antibody to mouse CD3ϵ (145-2C11, BD Pharmingen, San Diego, USA) and TCRβ(H57-597, BD Pharmingen, San Diego, USA); BUV563-conjugated antibody to mouse CD4 (GK1.5, BD Pharmingen, San Diego, USA); BUV737-conjugated antibody to mouse CD8α (53-6.7, BD Pharmingen, San Diego, USA).

### Blood cell count

Fresh blood samples were collected from the posterior orbital venous plexus of the mice in a heparin-containing polypropylene tube. The whole blood count was enumerated using Automated Hematology Analyzer (XT-1800i, Sysmex).

### *In vivo* cell depletion

For depletion of CD8^+^ T cells, mice were given an intraperitoneal injection of 200 μg mAb against CD8β (53-5.8, Bio X Cell, Lebanon, USA) 72 h before challenge, and after challenge, the antibodies were injected once weekly. For depletion of NK cells, anti-ASGM1 was injected intravenously 72 h before challenge, and after challenge, the antibody was injected every 7 d.

### CALR expression analysis

After treatment with oxaliplatin (10μM or 100 μM) or cisplatin (100 μM) for 4 h, CT26 cells were collected, washed twice with PBS and fixed in 0.25% paraformaldehyde in PBS for 5 min. After washing again twice in cold PBS, cells were incubated with the anti-calreticulin antibody (Abcam, ab2907) for 30 min at 4 °C, diluted in cold blocking buffer (2% fetal bovine serum in PBS), followed by washing and incubation with Alexa Fluor Plus 488-conjugated goat anti-rabbit antibody (A32731; Thermo Fisher Scientific) for 30 min at 4 °C. Each sample was then analyzed by flow cytometry on a FACS Celesta flow cytometer (BD Biosciences). Isotype-matched IgG antibodies were used as a control. The data were analyzed using FlowJo software (Tree Star).

### Immunofluorescence

After treatment with oxaliplatin (10μM or 100 μM) or cisplatin (100 μM) for 4 h, CT26 cells were placed on ice, washed twice with PBS and fixed in 0.25% paraformaldehyde in PBS for 5 min. Then the cells were washed twice in PBS, and stained with primary antibodies against CALR (Abcam, ab2907) for 30 min at 4 °C. After three washes in cold PBS, the cells were incubated for 30 min with Alexa Fluor Plus 488-conjugated goat anti-rabbit antibody (A32731; Thermo Fisher Scientific). Subsequently, the cells were fixed with 4% paraformaldehyde for 20 min. All slides were stained with 4′,6-diamidino-2-phenylindole (DAPI, Thermo Fisher Scientific) for 4 min and mounted on coverslips in ProLong™ Gold antifade solution (Thermo Fisher Scientific). Slides were visualized using an LSM880 confocal laser scanning microscope (Zeiss, Oberkochen, Germany).

### Enzyme-linked immunosorbent assay

The supernatants of CT26 cells were collected after treating with oxaliplatin (10μM or 100 μM) or cisplatin (100 μM) for 24 h. HMGB1 proteins were measured using the mouse HMGB1 ELISA KiT (NBP2-62767, Novus), according to the manufacturer’s instructions.

### Statistical analysis

Statistical analyses were performed in GraphPad Prism (La Jolla, USA) using appropriate tests as indicated in the legends (unpaired two-tailed t test, paired two-tailed t test, one-way ANOVA followed by Tukey’s multiple comparisons test or two-way ANOVA), with significant differences marked in all figures. Significance levels were defined as ns (not significant, p > 0.05), * p < 0.05, ** p < 0.01, *** p < 0.001, and **** p < 0.0001.

## Result

### Low-dose oxaliplatin improves TIGIT blockade immunotherapy against colon tumors

**Figure 1:**
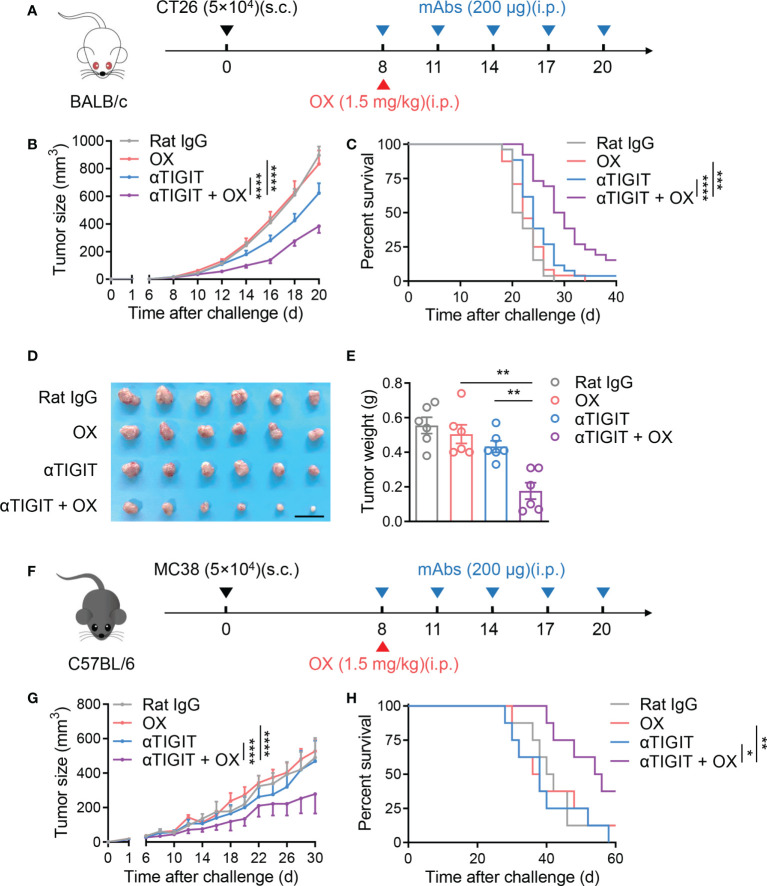
Low-dose oxaliplatin improves colon cancer immunotherapy of anti-TIGIT mAb. **(A)**, Experimental scheme for CT26 colon cancer model used in **(B–E)**. Mice were given injection of Rat IgG, anti-TIGIT mAb (10 mg/kg), oxaliplatin (OX, 1.5 mg/kg) or anti-TIGIT mAb plus OX intraperitoneally (i.p.) at various times after injection of 5×10^4^ CT26 tumor cells subcutaneously (s.c.) on day 0. **(B)**, Tumor size measurement at each time point. (n=16-20 mice per group). **(C)**, Overall survival of CT26-bearing mice with various treatments. **(D)**, Representative photograph and **(E)** weight of tumor (*n* = 6 per group) on day 21 after challenge. Scale bar represents 2 cm. **(F)**, Experimental scheme for MC38 colon cancer model used in **(G, H)**. Mice were given injection of Rat IgG, anti-TIGIT mAb, OX or anti-TIGIT mAb plus OX intraperitoneally (i.p.) at various times after injection of 5×10^4^ MC38 tumor cells subcutaneously (s.c.) on day 0. **(G)**. Tumor size measurement at each time point. (n=6-8 mice per group) **(H)**, Overall survival of MC38-bearing mice with various treatments. Data were representative of at least two independent experiments. Error bars represent means ± SEM. Statistical significance was determined using two-way ANNOVA **(B, G)**, Mantel–Cox test **(C, H)** or one-way ANOVA followed by Tukey’s multiple-comparisons **(E)**. *p < 0.05; **p < 0.01; ***p < 0.001 and ****p < 0.0001.

In the previous reports, OX and TIGIT blockade were recommended for locally advanced or metastatic colon cancer, respectively (26, 27). Here, we investigated the synergistic antitumor response of OX and anti-TIGIT mAb in MSS CRC. BALB/c mice were subcutaneously injected with murine MSS CT26 colon tumor cells (28), followed by treatment with rat IgG, anti-TIGIT mAb (10 mg/kg), OX (1.5 mg/kg) or anti-TIGIT mAb plus OX on day 8 post-tumor challenge (**Figure 1A**). In our setting, OX alone did not exert any therapeutic effect; however, as expected, anti-TIGIT mAb alone showed a slight inhibition of tumor growth but failed to extend the survival of CT26-bearing mice. Although OX monotherapy was inefficient, it could synergize with anti-TIGIT immunotherapy. Combination therapy with anti-TIGIT and OX suppressed tumor growth and significantly increased survival (**Figures 1B, C**). Smaller volumes and weights of tumors were observed on day 21 after tumor challenge in mice treated with anti-TIGIT and OX (**Figures 1D, E**). These phenomena were further verified in MC38-bearing mice (**Figure 1F**). Combination therapy with anti-TIGIT and OX delayed tumor growth and prolonged overall survival significantly; nevertheless, anti-TIGIT or OX monotherapy was inefficacy (**Figures 1G, H**).

**Figure 2:**
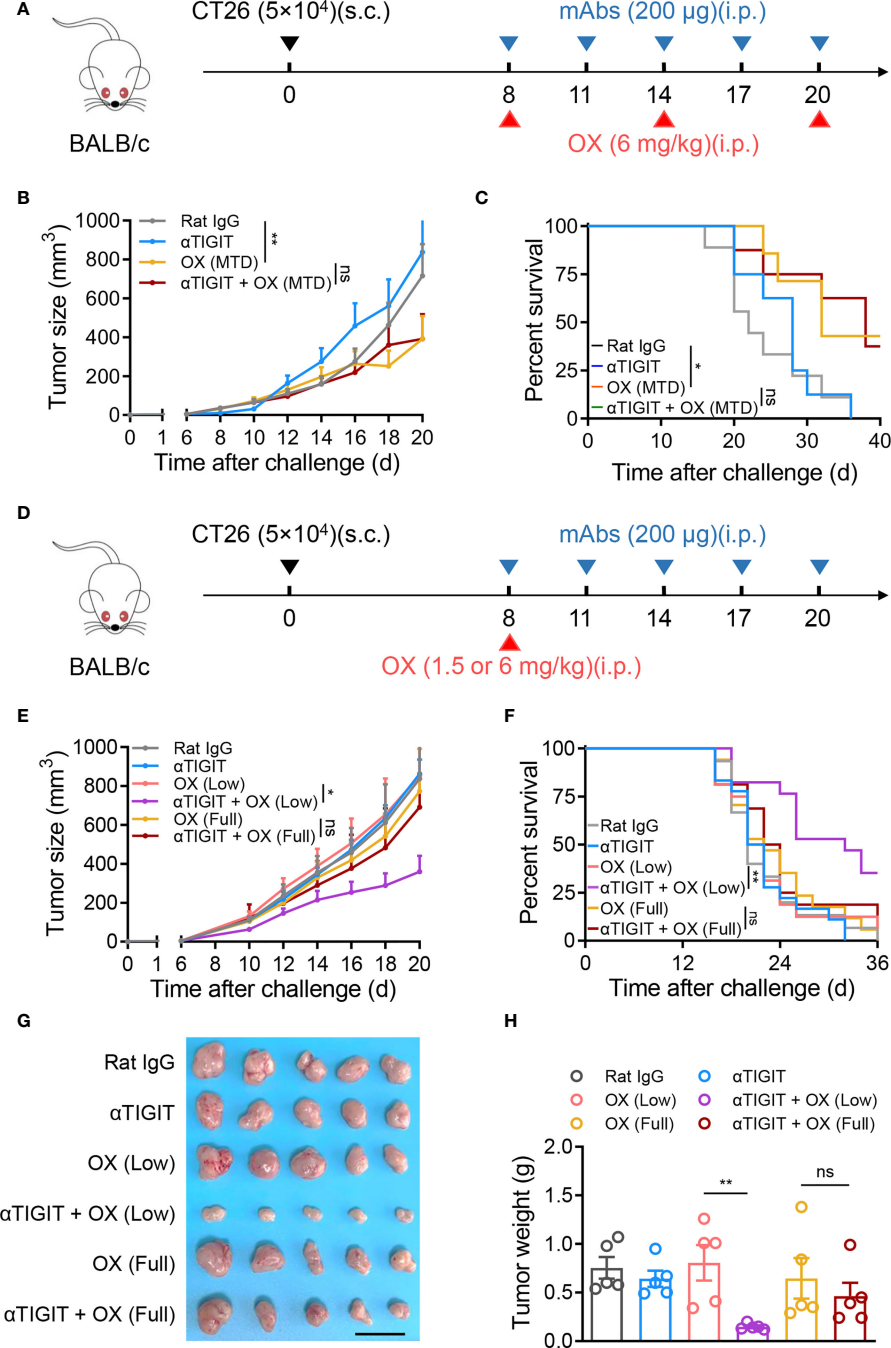
No synergistic antitumor activity of full-dose oxaliplatin and anti-TIGIT mAb. **(A)**, Experimental scheme for CT26 colon cancer model used in **(B, C)**. Mice were given injection of Rat IgG, anti-TIGIT mAb (10 mg/kg), OX (6 mg/kg), or anti-TIGIT mAb combined with OX intraperitoneally (*i.p.*) at various times after injection of 5×10^4^ CT26 cells subcutaneously (*s.c.*) on day 0. **(B)**, Tumor size measurement at each time point. (n=6-8 mice per group). **(C)**, Overall survival of CT26-bearing mice with various treatments. **(D)**, Experimental scheme for CT26 colon cancer model used in **(E–H)**. Mice were given injection of Rat IgG, anti-TIGIT mAb, various-dose OX or anti-TIGIT mAb combined with various-dose OX intraperitoneally (i.p.) at various times after injection of 5×10^4^ CT26 cells subcutaneously (s.c.) on day 0. **(E)**, Tumor size measurement at each time point. (n=6-8 mice per group). **(F)**, Overall survival of CT26-bearing mice with various treatments. **(G)**, Representative photograph and **(H)** weight of tumor (n = 5 per group) on day 21 after challenge. Scale bar represents 2 cm. Data were representative of at least two independent experiments. Error bars represent means ± SEM. Statistical significance was determined using two-way ANNOVA **(B, E)**, Mantel–Cox test **(C, F)** or one-way ANOVA followed by Tukey’s multiple-comparisons **(H)**. ns, p > 0.05; *p < 0.05 and **p < 0.01.

Platinum-based MTD chemotherapy has long been used as a first-line tumor therapy, including combination with ICB therapy. To assess whether MTD OX could enhance the efficacy of TIGIT blockade, intraperitoneal injection of OX at a dose of 6 mg/kg was repeated every 6 days for a total of 3 cycles with or without anti-TIGIT mAb (**Figure 2A**). Although MTD OX reduced tumor growth and increased overall survival in CT26-bearing mice, as expected, it failed to synergize with anti-TIGIT therapy (**Figures 2B, C**). We hypothesized that OX, as a chemotherapeutic drug, induced severe immunosuppression even if it could suppress the proliferation of tumor cells by itself. To verify this, the injection frequency of OX was decreased from 3 to 1 (**Figure 2D**). We observed that a single injection of full-dose OX (6 mg/kg) could not reduce tumor growth. Moreover, the synergistic antitumor effects of the combination therapy were not observed (**Figures 2E, F**). When the dose of oxaliplatin was reduced from 6 mg/kg to 1.5 mg/kg, the synergism of the combination treatment was observed; nevertheless, a single injection of low-dose OX or anti-TIGIT could not delay tumor growth or increase overall survival (**Figures 2E, F**). Decreased tumor size and weight were also observed on day 21 after tumor challenge in mice treated together with anti-TIGIT mAb and single-injection low-dose OX (**Figures 2G, H**). These results suggest that single-injection low-dose OX, but not full-dose OX, was able to synergize with anti-TIGIT immunotherapy against colon tumors.

### Combination treatment with low-dose oxaliplatin and TIGIT elicits an active tumor-immune microenvironment

**Figure 3:**
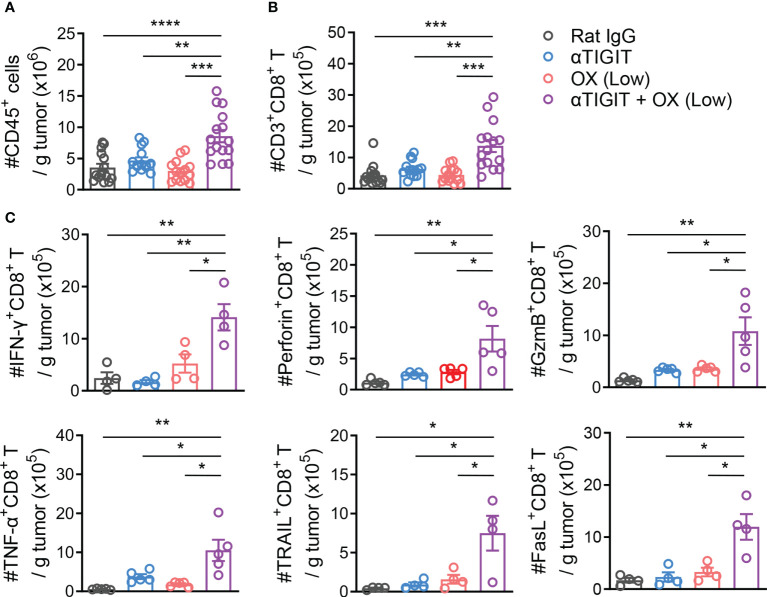
Combination treatment with Low-dose Oxaliplatin and TIGIT blockade Increases Tumor Infiltration and Activation of CD8^+^ T Cells. **(A)**, CT26-bearing mice received various treatments or were left untreated. On day 21 after challenge, tumors were harvested and tumor-infiltrating lymphocytes were isolated. Absolute numbers of tumor-infiltrating CD45^+^ T cells were measured by flow cytometry. **(B)**, Absolute numbers of CD3^+^CD8^+^ T cells measured by flow cytometry from mice as in **(A)**. **(C)**, Absolute numbers of tumor-infiltrating CD8^+^ T expressing IFN-γ, Perforin, Granzyme B, TNF-α, TRAIL and FasL measured by flow cytometry from mice as in **(A)**. Each symbol represents an individual mouse. Data were representative of at least two independent experiments. Error bars represent means ± SEM. Statistical significance was determined using one-way ANOVA followed by Tukey’s multiple-comparisons **(A–C)**. *p < 0.05; **p < 0.01; ***p < 0.001 and ****p < 0.0001.

To explore the possible mechanisms of combination therapy, we compared the numbers and activation of tumor-infiltrating lymphocytes (TILs) in CT26-bearing mice with various treatments. Mice were sacrificed at day 21 after tumor challenge, and TILs were isolated and analyzed. The absolute number of CD45^+^ TILs increased significantly in mice treated with anti-TIGIT mAb plus low-dose OX (**Figure 3A**). Furthermore, combination therapy also increased the absolute number of tumor-infiltrating CD8^+^ T cells (**Figure 3B**). Tumor-bearing mice treated with combination therapy showed higher amounts of CD8^+^ TILs that expressed IFN-γ, Granzyme B, Perforin, TNF-α, TRAIL and FasL than untreated mice, OX or TIGIT mAb alone (**Figure 3C**). These results suggested that combination therapy with TIGIT blockade and low-dose oxaliplatin increased the numbers and activation of CD8^+^ TILs, induced an active tumor-immune microenvironment.

**Figure 4:**
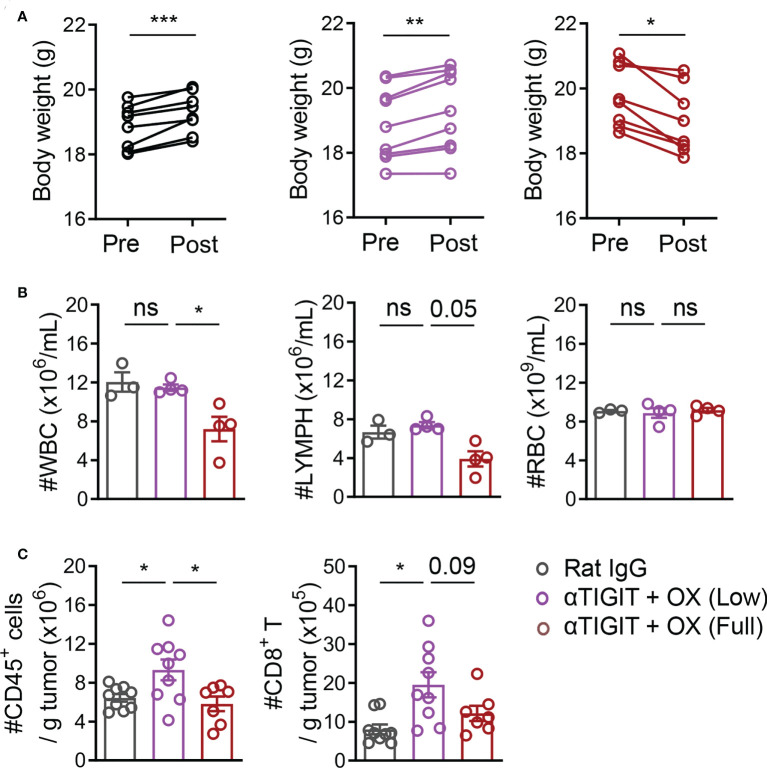
Full-dose oxaliplatin induces greater immunosuppression. **(A)**, Body weight in CT26-bearing mice before or after treatment with Rat IgG, anti-TIGIT mAbs plus various-dose OX for 2 days. **(B)**, The counts of WBC (left), Lymph (mid) and RBC (right) in peripheral blood of CT26-bearing mice. **(C)**, Absolute numbers of tumor-infiltrating CD45^+^ cells and CD8^+^ T cells. Each symbol represents an individual mouse. Data were representative of at least two independent experiments. Error bars represent means ± SEM. Statistical significance was determined using paired two-tailed t test **(A)** or one-way ANOVA followed by Tukey’s multiple-comparisons **(B, C)**. ns, p > 0.05; *p < 0.05; **p < 0.01 and ***p < 0.001.

To investigate the reason why full-dose OX and TIGIT blockade had no synergism, CT26-bearing mice were treated with full-dose (6 mg/kg) or low-dose (1.5 mg/kg) OX, together with anti-TIGIT mAb. In the mice that received full-dose (6 mg/kg) OX treatment with or without anti-TIGIT mAb for 2 days, significant reductions in body weight, white blood cells (WBCs) and peripheral blood lymphocytes were observed (**Figures 4A, B**). The absolute numbers of CD45^+^ TILs and CD8^+^ TILs were also decreased significantly (**Figure 4C**). In contrast, the body weights were moderately increased in tumor-bearing mice treated with 1.5 mg/kg OX (**Figure 4A**). The numbers of white blood cells, peripheral blood lymphocytes, CD45^+^ TILs and CD8^+^ TILs were similar to those in untreated mice (**Figures 4B, C**). These findings indicated that full-dose OX, but not low-dose OX, induced severe immunosuppression and impaired the efficacy of combination therapy.

### Synergistic efficacy of low-dose oxaliplatin and TIGIT blockade depends on CD8^+^ T

**Figure 5:**
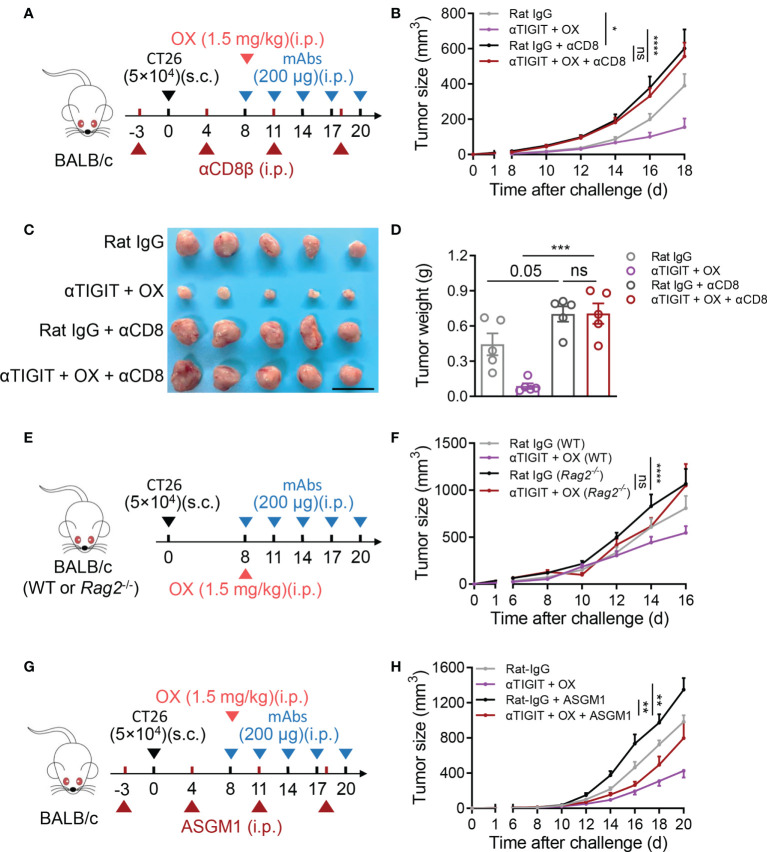
Deficiency of CD8^+^ T cells impairs synergistic antitumor efficacy of low-dose oxaliplatin and anti-TIGIT. **(A)**, Experimental scheme for CT26 colon cancer model used in **(B–D)**. Mice were given injection of Rat IgG or anti-TIGIT (10 mg/kg) combined with OX (1.5 mg/kg) intraperitoneally (i.p.) at various times after injection of 5×10^4^ CT26 cells subcutaneously (s.c.) on day 0 and weekly injection of anti-CD8β mAbs intraperitoneally (i.p.) on day -3. **(B)**, Tumor size measurement at each time point (n = 9 mice per group). **(C)**, Representative photograph and **(D)** weight of tumor on day 18 after challenge. Scale bar represents 2 cm. (n = 9 mice per group). **(E)**, Experimental scheme for CT26 colon tumor model used in **(F)** BALB/c WT or BALB/c *Rag2^-/-^* mice were given injection of Rat IgG or anti-TIGIT (10 mg/kg) combined with OX (1.5 mg/kg) intraperitoneally (i.p.) at various times after injection of 5×10^4^ CT26 cells subcutaneously (s.c.) on day 0. **(F)**, Tumor size measurement at each time point (n = 9 mice per group). **(G)**, Experimental scheme for CT26 colon tumor model used in **(B)** Mice were given injection of Rat IgG or anti-TIGIT (10 mg/kg) combined with OX (1.5 mg/kg) intraperitoneally (i.p.) at various times after injection of 5×10^4^ CT26 cells subcutaneously (s.c.) on day 0 and weekly injection of anti-ASGM1 intraperitoneally (i.p.) on day -3. **(H)**, Tumor size measurement at each time point (n = 8-10 mice per group). Data were representative of at least two independent experiments. Error bars represent means ± SEM. Statistical significance was determined using two-way ANNOVA **(B, F, G)**, one-way ANOVA followed by Tukey’s multiple-comparisons **(D)**. ns, p > 0.05; *p < 0.05; **p < 0.01; ***p < 0.001 and ****p < 0.0001.

To further investigate the roles of CD8^+^ T and NK cells in TIGIT blockade combined with low-dose OX, CD8^+^ T cells or NK cells in tumor-bearing mice were depleted by treatment with anti-CD8β or anti-ASGM1, respectively (**Figures 5A, G**). As expected, the deficiency of CD8^+^ T cells significantly led to accelerated tumor growth, including tumor size (**Figure 5B**), tumor volume (**Figure 5C**) and tumor weight (**Figure 5D**). TIGIT blockade combined with low-dose OX lost the inhibition of tumor growth in CD8^+^ T deficient mice compared to that in untreated mice (**Figure 5D**). Additionally, in *Rag2^–/–^* mice (**Figure 5E**), combination treatment did not reveal synergistic efficacy on CT26 tumors (**Figure 5F**). The reverse results were observed in the NK-cell-depleted mice, where combination treatment could control tumor growth (**Figure 5H**). In other words, there were some synergies between low-dose OX and anti-TIGIT mAb, even if the absence of NK cells. These findings indicated that the therapeutic efficacy of combination treatment depends on CD8^+^ T cells.

### Lack of synergy between nonimmunogenic chemotherapeutics and TIGIT blockade

**Figure 6:**
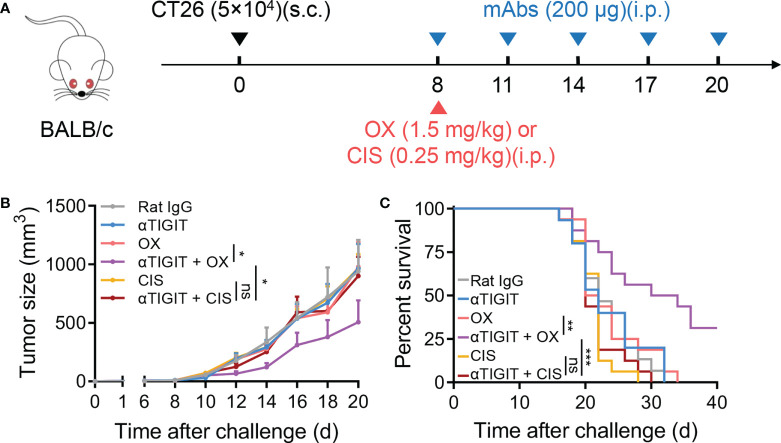
Low-dose cisplatin fails to promote anti-TIGIT mAb treatment against CT26 colon cancer. **(A)**, Experimental scheme for CT26 colon cancer model used in **(B, C)**: mice were given injection of Rat IgG, anti-TIGIT (10 mg/kg), OX (1.5 mg/kg), anti-TIGIT combined with OX, cisplatin (CIS, 0.25 mg/kg) or anti-TIGIT combined with CIS intraperitoneally (*i.p.*) after injection of 5×10^4^ CT26 tumor cells subcutaneously (*s.c.*) on day 0. **(B)**, Tumor size measurement at each time point. (n=15-17 mice per group). **(C)**, Overall survival of CT26-bearing mice with various treatments. (n=7 or 8 mice per group). Data were representative of at least two independent experiments. Error bars represent means ± SEM. Statistical significance was determined using two-way ANNOVA **(B)** or Mantel–Cox test **(C)**. ns, p > 0.05; *p < 0.05; **p < 0.01 and ***p < 0.001.

Finally, we investigated whether other low-dose chemotherapeutic drugs could also synergize with TIGIT blockade. Cisplatin (CIS), a platinum-based drug, has been used in the treatment of various types of tumors including colon cancer. In our study, CT26-bearing mice were treated with low-dose cisplatin and anti-TIGIT mAb on day 8 posttumor challenge (**Figure 6A**). We found that CIS treatment alone provided minimal control of CT26 tumor progression, similar to OX treatment. Furthermore, low-dose CIS treatment combined with anti-TIGIT mAb did not delay tumor progression or increase overall survival (**Figures 6B, C**). These results suggested that OX, but not CIS, could synergize with TIGIT blockade, even if they were both platinum-based drugs.

**Figure 7:**
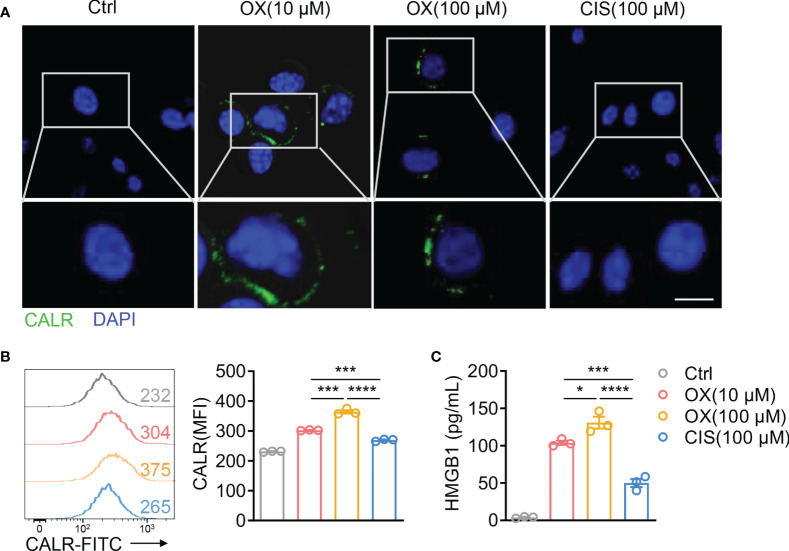
Oxaliplatin induces immunogenic cell death of CT26 colon cancer. **(A)**, Representative immunofluorescence staining of calreticulin (CALR) in CT26 cells after treatment with various dose OX or CIS for 4 h. Scale bar represents 10 μm. **(B)**, Representative histogram of CALR expression on CT26 cells after treatment with various dose OX or CIS for 4 h (left). Mean fluorescent intensity (MFI) of CALR expression on CT26 were also shown (right). **(C)**, HMGB1 concentration in the supernatants of CT26 cells treated with various dose OX or CIS for 24 h. Data were representative of at least two independent experiments. Error bars represent means ± SEM. Statistical significance was determined using unpaired two-tailed t-test **(B, C)**. *p < 0.05; ***p < 0.001 and ****p < 0.0001. .

Considering that low-dose OX, but not CIS, showed synergistic antitumor effects with TIGIT blockade, we hypothesized that low-dose OX sensitized colon tumors to checkpoint blockade therapy. Therefore, CT26 cells were treated with various dose OX or CIS *in vitro*. High mobility group box 1 (HMGB1) release and calreticulin (CALR) exposure were used as surrogate markers for drug-induced immunogenic death (29). After 4 h of stimulation, CT26 cells treated with OX, but not CIS, exposed CALR on the cell surface as determined by immunofluorescence staining (**Figure 7A**) and flow cytometric analysis (**Figure 7B**), and released higher levels of HMGB1 (**Figure 7C**). These data demonstrated that OX could induce immunogenic death of tumor cells, which might sensitize tumors to TIGIT blockade therapy.

## Discussion

In recent years, combination therapies of ICB and chemotherapeutics have been clinically approved for various tumors. Nevertheless, many patients have no more benefit from combination therapies than chemotherapy alone (30–35). In this study, we found that low-dose OX, combined with TIGIT blockade, triggered a synergistic antitumor response in CT26-bearing mice. In contrast, full-dose OX induced more severe immunosuppression and impaired the efficacy of combination therapy. In addition, we reported that selected immunogenic chemotherapeutics could sensitize colon cancer to ICB therapy. The synergistic antitumor response initiated by the immunogenic chemotherapeutics depended on CD8^+^ T cells.

Despite severe side effects, MTD chemotherapy regimen is still a standard therapy of various tumors (36). Therefore, MTD chemotherapy is also the top choice of combination therapy based on ICB. For colon cancer, FOLFOX (5-fluorouracil, L-leucovorin plus oxaliplatin) and XELOX (oxaliplatin plus capecitabine) regimens were clinically approved in the 2000s (37). These chemotherapeutic drugs interfere with cell proliferation by targeting DNA/RNA synthesis and cellular metabolism. Since they have no specificity, these drugs impair not only tumor cells but also lymphocytes. On the other hand, the efficacy of ICB therapy depends on the tumor infiltration of lymphocytes (38). Lymphopenia, the common side effect of MTD chemotherapy, limits the outcome of ICB. This is a possible reason why the patients did not benefit more from combination treatment of chemotherapy and ICB. In this study, we tried to improve the efficacy of combination treatment by reducing the toxicity of chemotherapy. We found that low-dose OX could not induce body weight loss or lymphocyte decrease. Importantly, it was able to sensitize tumor cells to TIGIT blockade therapy, although low-dose OX alone failed to inhibit the growth of tumor cells. These findings suggested that the dosage or regimen of chemotherapy should be optimized when it combined with ICB therapy. The balance of tumor cell sensitization and immunosuppression requires further investigation.

Due to the lack of lymphocyte infiltration, pMMR/MSS CRC patients are resistant to ICB therapy (39). Chemotherapy, as the first-line treatment for pMMR/MSS CRC patients, was chosen to overcome this resistance. Chemotherapeutic drugs reduce the growth of tumor cells by inducing cell death (40). The predominant type of drug-induced cell death is apoptosis, which is frequently nonimmunogenic or tolerogenic. Thus, most chemotherapeutic drugs fail to boost antitumor immune responses. However, several drugs, such as oxaliplatin and doxorubicin, induce immunogenic tumor cell death and increase CD8^+^ T-cell infiltration by releasing antigens and causing inflammation in the tumor microenvironment (41). These agents might transform ‘‘cold’’ tumors into immunologic ‘‘hot’’ environments and reverse resistance to ICB in pMMR/MSS CRC patients. We found that OX sensitized CT26 tumors, which was regarded as a MSS CRC and resistant to ICB, to TIGIT blockade by improving CD8^+^ T-cell infiltration. In contrast, CIS, which is a non-ICD-induced platinum-based drugs, failed to overcome this resistance. Our study indicated that the appropriate selection of drugs determined the efficacy of ICB for MSS CRC treatment. The majority of approved chemotherapeutic drugs might not synergize with ICB therapy.

In summary, our findings provided two pieces of evidence that 1) chemotherapeutic drugs should be selected for their ability to induce immunogenicity in tumors and provide synergistic benefits when combined with ICB therapy, and 2) they should be reduced to an appropriate dosage that could sensitize tumors to ICB therapy but not induce immunosuppression. These findings need to be further confirmed in various tumor models. Despite this limitation, our data suggested that the strategies of combination treatment of MSS CRC with chemotherapy and ICB should be re-evaluated. The chemotherapeutic drugs should be chosen for the potential to induce ICD and the dosage and regimen should also be optimized.

## Data availability statement

The original contributions presented in the study are included in the article/supplementary material. Further inquiries can be directed to the corresponding authors.

## Ethics statement

The animal study was approved by the ethics committee of the University of Science and Technology of China.

## Author contributions

YF, RS, XZ designed the study. YF, XX and MT performed the experiments and analyses. ZT, HS and HW provided advice. YF and XZ wrote and revised the manuscript. ZT, HW, RS, HS and XZ supervised the study. RS and XZ critically reviewed the manuscript. All authors contributed to the article and approved the submitted version.

## Funding

This work was supported by National Key R&amp;D Program of China (2019YFA0508502/3, 2018YFA0507403), Strategic Priority Research Program of the Chinese Academy of Science (XDB29030201) and Natural Science Foundation of China (#81788101).

## Conflict of interest

The authors declare that the research was conducted in the absence of any commercial or financial relationships that could be construed as a potential conflict of interest.

## Publisher’s note

All claims expressed in this article are solely those of the authors and do not necessarily represent those of their affiliated organizations, or those of the publisher, the editors and the reviewers. Any product that may be evaluated in this article, or claim that may be made by its manufacturer, is not guaranteed or endorsed by the publisher.

## Abbreviations

CRC, colorectal cancer; ICB, immune checkpoint blockade; OX, oxaliplatin; CIS, cisplatin; TIL, tumor-infiltrating lymphocyte; ICD, immunogenic cell death; MTD, maximum tolerated dose; dMMR, mismatch repair deficient; MSI, microsatellite instability; pMMR, mismatch repair proficient; MSS, microsatellite stability; mAb, monoclonal antibody; WBC, white blood cell; LYMPH, lymphocyte; RBC, red blood cell; DAPI, 4′,6-diamidino-2-phenylindole; MFI, mean fluorescent intensity, CALR, calreticulin; HMGB1, high mobility group box 1.
